# Regulatory T cells with a defect in inhibition on co-stimulation deteriorated primary biliary cholangitis

**DOI:** 10.18632/oncotarget.22658

**Published:** 2017-11-26

**Authors:** Jianing Chen, Xianliang Hou, Hongyu Jia, Guangying Cui, Zhongwen Wu, Lin Wang, Chong Lu, Wei Wu, Yingfeng Wei, Toshimitsu Uede, Lanjuan Li, Zhexiong Lian, Hongyan Diao

**Affiliations:** ^1^ State Key Laboratory for Diagnosis and Treatment of Infectious Diseases, Collaborative Innovation Center for Diagnosis and Treatment of Infectious Diseases, The First Affiliated Hospital, College of Medicine, Zhejiang University, Hangzhou, China; ^2^ Molecular Immunology, Institute for Genetic Medicine, Hokkaido University, Sapporo, Japan; ^3^ Liver Immunology Laboratory, Institute of Immunology and The CAS Key Laboratory of Innate Immunity and Chronic Disease, School of Life Sciences, University of Science and Technology of China, Hefei, China

**Keywords:** primary biliary cholangitis, regulatory T cell, proliferation, co-stimulation, Immunology and Microbiology Section, Immune response, Immunity

## Abstract

Regulatory T cells (Tregs) play an indispensable role in the progression of primary biliary cholangitis (PBC). Although Tregs could normalize costimulation in *in vivo* and *in vitro* models, it is obscure whether and how Tregs mediate these effects in PBC. Herein we focused on the quantitative and functional characteristics of Tregs in PBC. The number and proportion of Tregs, and the production of interleukin (IL)-10 were all significantly less in the PBC patients than in the healthy controls (HCs). In addition, compared to the HCs, the costimulatory CD86 of the circulation and liver were significantly higher in the patients with PBC. CD86 expression on CD1c^+^ cells negatively correlated with the proportion of Tregs. There was also a positive correlation between mayo risk score and the ratio of CD86/Treg. *In vitro* experiments showed that inhibition of CD86 expression on CD1c^+^ cells by Tregs was significantly weakened in the PBC patients. Furthermore, the autoantibodies from the PBC patients could promote CD86 expression on CD1c^+^ cells and transforming growth factor-β production by human hepatic stellate cells. Overall, Tregs declined in inhibition on co-stimulation expression in the presence of autoantibodies, which could be associated to PBC-related bile duct injury and fibrosis. This indicated that maintenance of balance of co-stimulation and Tregs could be beneficial for PBC.

## INTRODUCTION

Primary biliary cholangitis (PBC) is a chronic impairment of bile flow characterized by progressive destruction of small intrahepatic bile ducts and liver inflammation. This can lead to liver cirrhosis and ultimately require liver transplantation for survival. The specific presence of antimitochondrial antibodies in serum is an important indicator of PBC [1, 2], as well as activation of quiescent human stellate cells that develop a myofibroblast-like phenotype. The latter proliferate and produce intermediate filaments and α-smooth muscle actin (α-SMA) that leads to fibrosis at the advanced stage. Although there have been investigational and clinical improvements in PBC such as ursodeoxycholic acid therapy [3], there is still much that is unknown regarding the pathogenesis of this disease.

Maintainance of regulatory T cells (Tregs) in quantity or function,is important for control of the immune response. Treg function may suppress autoreactive lymphocyte proliferation and cytokine production via contact with target cells or the release of immunoregulatory cytokines [4]. It was reported that colitis and primary sclerosing cholangitis develop spontaneously in Interleukin (IL) -2Rα-deficient mice, a model that is characterized by Treg dysfunction [5]. In addition, defective regulation by Tregs was observed in a significant proportion of patients with systemic or organ-specific autoimmune diseases [6-8].

It was also reported that the co-stimulatory CD86 positive cells might be involved in antigen presentation to helper T cells infiltrating the periductal tissue in the PBC-related bile duct injury [9]. Tregs were also implicated in the regulation of dendritic cell (DC) co-stimulation CD80 and CD86 *in vivo*, which preferentially promoted the responses and interactions of T effector cells. In mice the reconstitution of Tregs during lymphopenia could normalize DC co-stimulation to control the proliferation of T lymphocytes [10]

We hypothesized that Treg function is compromised in PBC and may be related to alterations in T cell immunobiology as well as co-stimulatory. The mechanism by which Tregs exert their suppressor or regulatory activity has not been definitively characterized. Our study investigated the regulation of T cells and co-stimulation by Tregs in the patients with PBC.

## RESULTS

### General characteristics of the PBC patients

We compared the clinical characteristics of the PBC patients and the HCs. The two groups were age- and gender- matched. The albumin levels of the two groups were similar. The aminotransferase, ALP, γ-GT and bilirubin levels were significantly higher in the PBC patients than the HCs. Of the PBC patients, 74.5% were positive for antimitochondrial antibodies. The Mayo risk score of the PBC patients was also higher than that of the HCs (Table 1).

**Table 1:** Demographic and clinical characteristics of the study groups

	HC	PBC	*P*
Age, median y	49	51	0.9148
Gender, n female/male	48/13	43/12	0.9472
Albumin, g/L	45.98 ± 2.336	40.73 ± 3.063	0.0926
Aspartate aminotransferase, U/L	20.45 ± 5.184	73.25 ± 7.578	<0.001
Alanine aminotransferase, U/L	18.63 ± 9.551	113.30 ± 2.847	<0.001
Alkaline phosphatase, U/L	58.67 ± 9.823	152.76 ± 23.323	<0.001
Glutamyl transpeptidase, U/L	20.19 ± 10.667	194.76 ± 42.078	<0.001
Total bilirubin, μmol/L	9.64 ± 4.279	24.41 ± 8.407	<0.001
Direct bilirubin, μmol/L	3.48 ± 1.383	14.79 ± 2.931	<0.001
Anti-mitochondrial antibody, n +/–	—	41/14	—
Mayo risk score	1.91 ± 0.486	4.86 ± 1.359	<0.001

**Figure 1:**
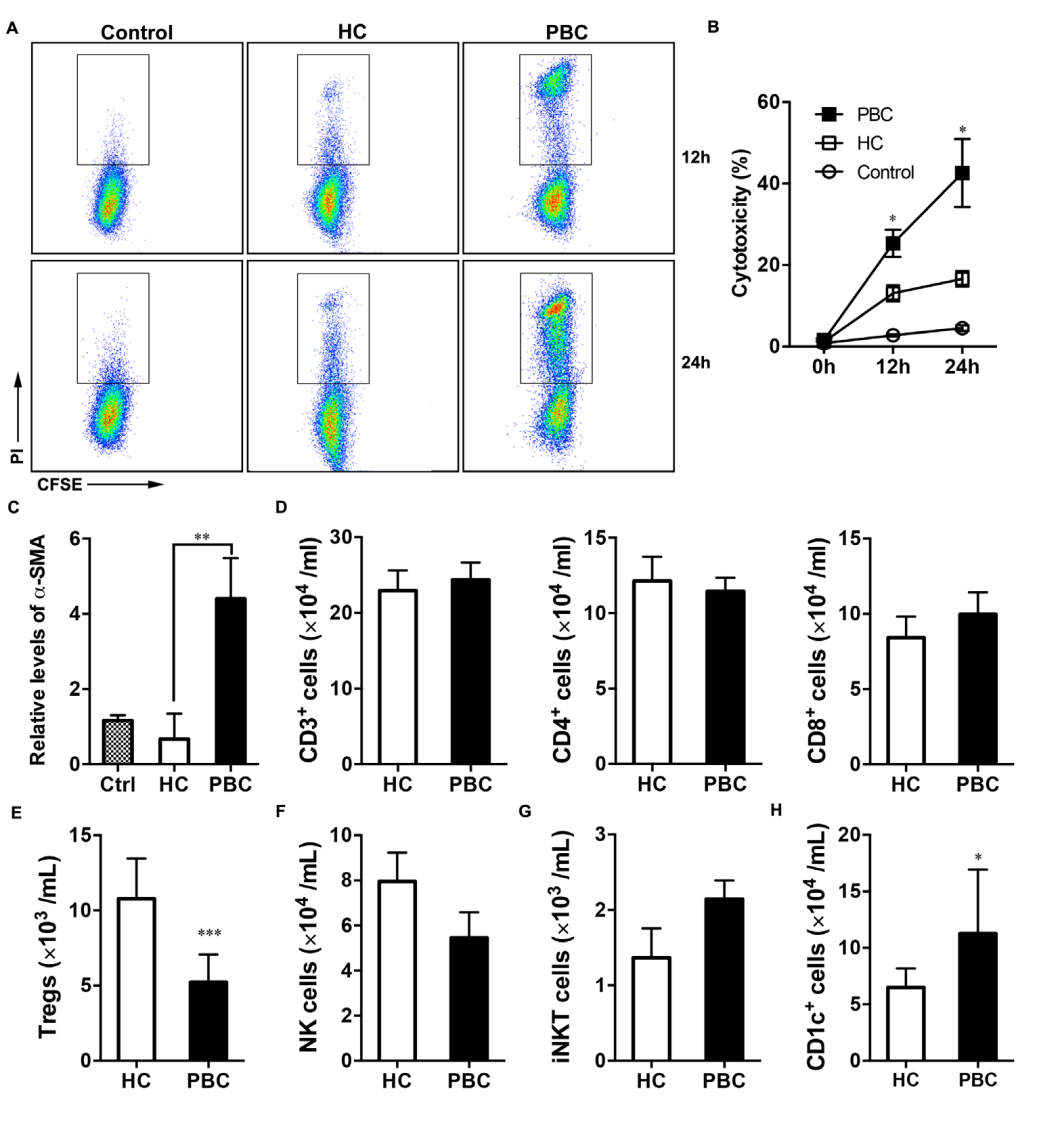
The fewer absolute number of Tregs in PBC patients (**A**) Cytotoxicity of PBMCs against human intrahepatic biliary epithelial cells (RBE) was shown in the PBC and HC groups. (**B**) Statistical data of the cytotoxicity against RBE (*n* = 8 per group). (**C**) Human stellate cell line (LX-2) was cultured with PBMCs from the PBC and HC groups (*n =* 6 per group). α-SMA expression by LX-2. The absolute numbers of lymphocytes of the PBC and HC groups were detected by flow cytometry (*n* = 55 and *n* = 61), including (**D**) CD3^+^, CD4^+^ and CD8^+^ cells. (**E**) Tregs (CD4^+^ CD25^high^ CD127^low^). (**F**) NK (CD3^–^ CD56^+^) cells. (**G**) iNKT cells. (**H**) CD1c^+^ cells. Mann-Whitney U test. Data shown are mean ± SD. *P < 0.05, **P < 0.01, ***P < 0.001.

Compared to the controls, histopathology revealed more inflammatory infiltration, damage of interlobular bile duct and more hepatic fibrosis (Supplementary Figure 1). Therefore, PBMCs-mediated cytotoxicity in the two groups was tested against a biliary epithelial cell line (i.e., RBE). The PBMCs of the PBC patients showed a notably higher cytotoxicity against cholangiocytes compared with that of the PBMCs of the HC group, and this was time-dependent (Figure 1A and 1B). Furthermore, the α-SMA expression of LX-2 cells cultured with PBMCs from the patients was > 4-fold that of the LX-2 cells cultured alone, whereas when cultured with PBMCs from HCs the level of α-SMA was only 0.5-fold higher (Figure 1C).

### Decreased numbers of Tregs in the PBC groups

To address the involvement in the bile duct damage and hepatic fibrosis, the absolute numbers of various lymphocytes in the peripheral blood were analyzed. The PBC patients and HC individuals were similar with regard to the analyzed T lymphocyte subpopulations (CD3^+^, CD4^+^, and CD8^+^; Figure 1D), and the absolute numbers of natural killer (NK) and iNKT cells were also similar between the two groups (Figure 1F and 1G). Interestingly, the absolute numbers of CD1c^+^ cells were markedly increased in the PBC patients than the HCs (Figure 1H).

**Figure 2:**
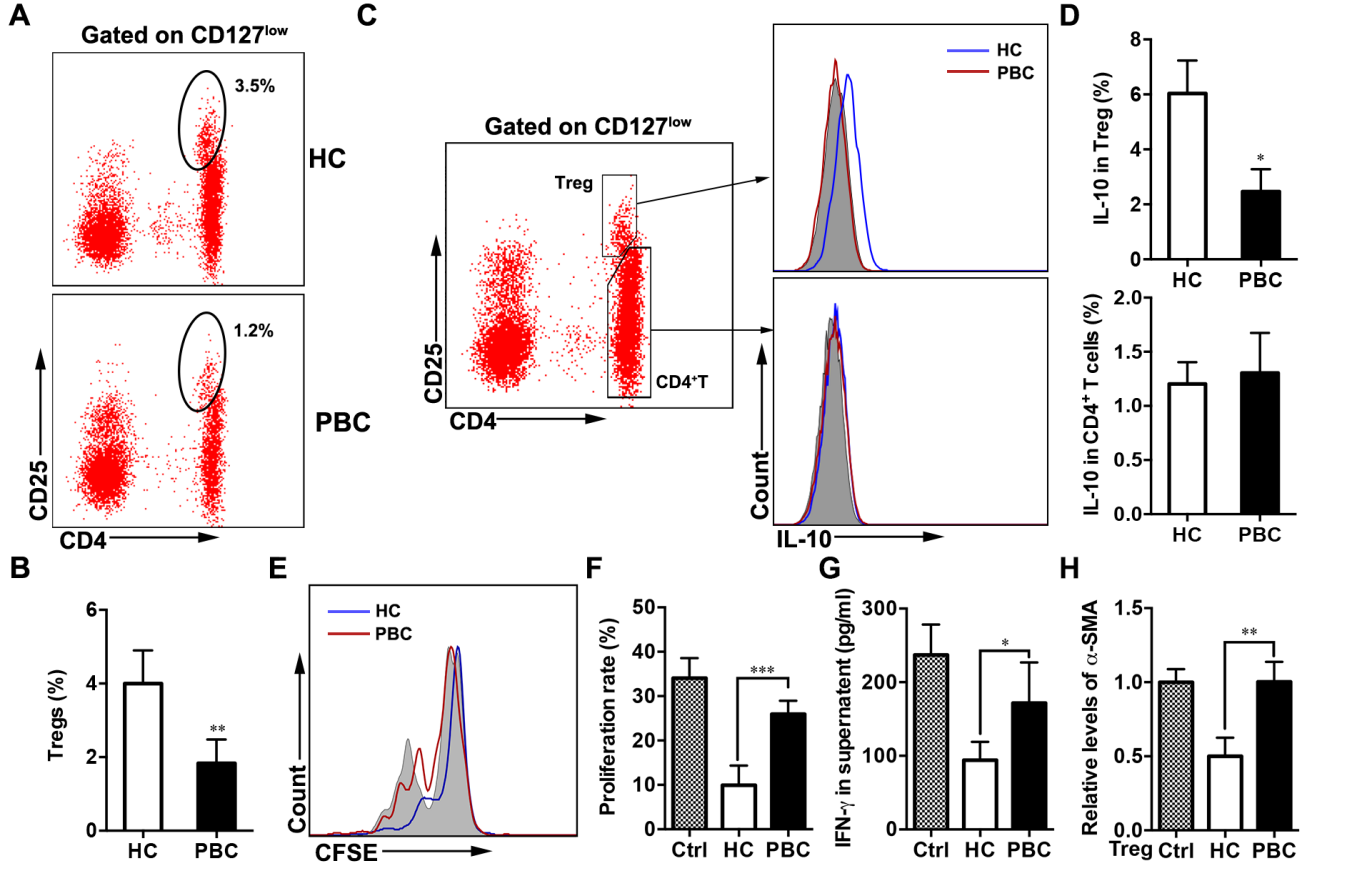
Lower proportion and deficient suppression of Tregs in PBC patients (**A**) Proportion of Tregs was determined by flow cytometry and compared between the PBC and HC groups, Representative FACS plots are shown; statistical data is shown in (**B**) (*n* = 55 and *n* = 61). Comparisons of intracellular IL-10 production by Tregs and CD4^+^ T cells between the PBC and HC groups, Representative image are shown in (**C**) and statistical data is shown in (**D**) (*n* = 10 per group), grey is isotype control. (**E**) CD4^+^ T cells were cultured with or without (grey) Tregs from the PBC and HC groups, respectively. (**F**) Inhibition of Tregs on CD4^+^ T cells and the statistical data (*n* = 8 per group). (**G**) The levels of IFN-γ were determined in the supernatant by ELISA (*n* = 8 per group). (**H**) And α-SMA expression by LX-2 was detected with treatment of Tregs with the two groups. Data shown are mean ± SD. Unpaired Student’s *t* test (A-D), ANOVAs (E-H). *P < 0.05, **P < 0.01, ***P < 0.001.

It has been reported that there may be impairment in the number of Tregs in other autoimmune diseases, such as inflammatory bowel disease [11]. Therefore, CD4^+^, CD25^high^, and CD127^low^ Tregs were analyzed in the present study. The PBC patients had significantly lower numbers of Tregs relative to the HC group (Figure 1D). Also, the proportion of Tregs was significantly lower in the PBC patients compared to the HCs (Figure 2A and 2B).

### Impaired immunosuppression ability of Tregs in the PBC group

Since IL-10 is postulated to function as a negative regulator in some autoimmune diseases [12], the intracellular IL-10 by CD4^+^ T cells and Tregs was also investigated. We found that in the PBC patients the ability to produce IL-10 by Tregs was clearly inferior to that of the HCs, while the production by CD4^+^ T cells of the two groups was not significantly different (Figure 2C and 2D). This suggested impairment in IL-10 production by Tregs activation in the patients with PBC.

The apparent impairment of Tregs was further addressed in PBC patients by quantitating and comparing the Treg functions of the two groups (Figure 2E-2H). We then isolated the Tregs from both groups for *in vitro* suppression assays against T effector (CD4^+^CD25^–^) cells from PBC patients. When tested against T effector cells, the Tregs of the HC group were significantly more immunosuppressive than that of the PBC patients (Figure 2E and 2F). This further suggested a deficiency in Treg regulation in PBC.

To further investigate the feeble immunosuppression of the PBC Tregs, we analyzed IFN-γ levels in supernatants of the above co-culture. IFN-γ produced in activated T cells or NK cells is involved in the inflammatory responses [13], which have been implicated in the cytotoxicity, with reference to the biliary epithelial cells, in the progression of PBC. IFN-γ secretion by the T effector cells was significantly inhibited when cultured with HC Tregs. However, the Tregs of the PBC patients did not effectively suppress the IFN-γ production by T effectors. The inhibition of IFN-γ production by the PBC Tregs was still far lower than that of the HCs (Figure 2G), and this is consistent with the results of the cytotoxicity assay.

IL-10 levels were also detected in the supernatant. As the intracellular IL-10 production by the HC Tregs was higher than that of the PBC group, we speculated that HC Tregs might protect against T cell-mediated biliary tract injuries via IL-10. We found that IL-10 secretion was markedly higher when cultured with HC Tregs than treatment with PBC Tregs (Supplementary Figure 2A). And the IL-10 levels in the supernatant with PBMCs from the PBC patients were significantly lower than that of the HCs (Supplementary Figure 2B). Also, inhibition on liver fibrosis could be IL-10 dependent, as shown that the expression of α-SMA by LX-2 cells was decreased when treated with IL-10 (Supplementary Figure 2C). As indicated above, a deficiency in inhibition on liver fibrosis in the PBC patients, the isolated Tregs from the two groups were each directly co-cultured with LX-2 cells. The expression of α-SMA by LX-2 cells was higher when cultured with Tregs from the PBC patients compared with that of the HCs (Figure 2H).

### Overexpression of co-stimulation in the PBC patients

**Figure 3:**
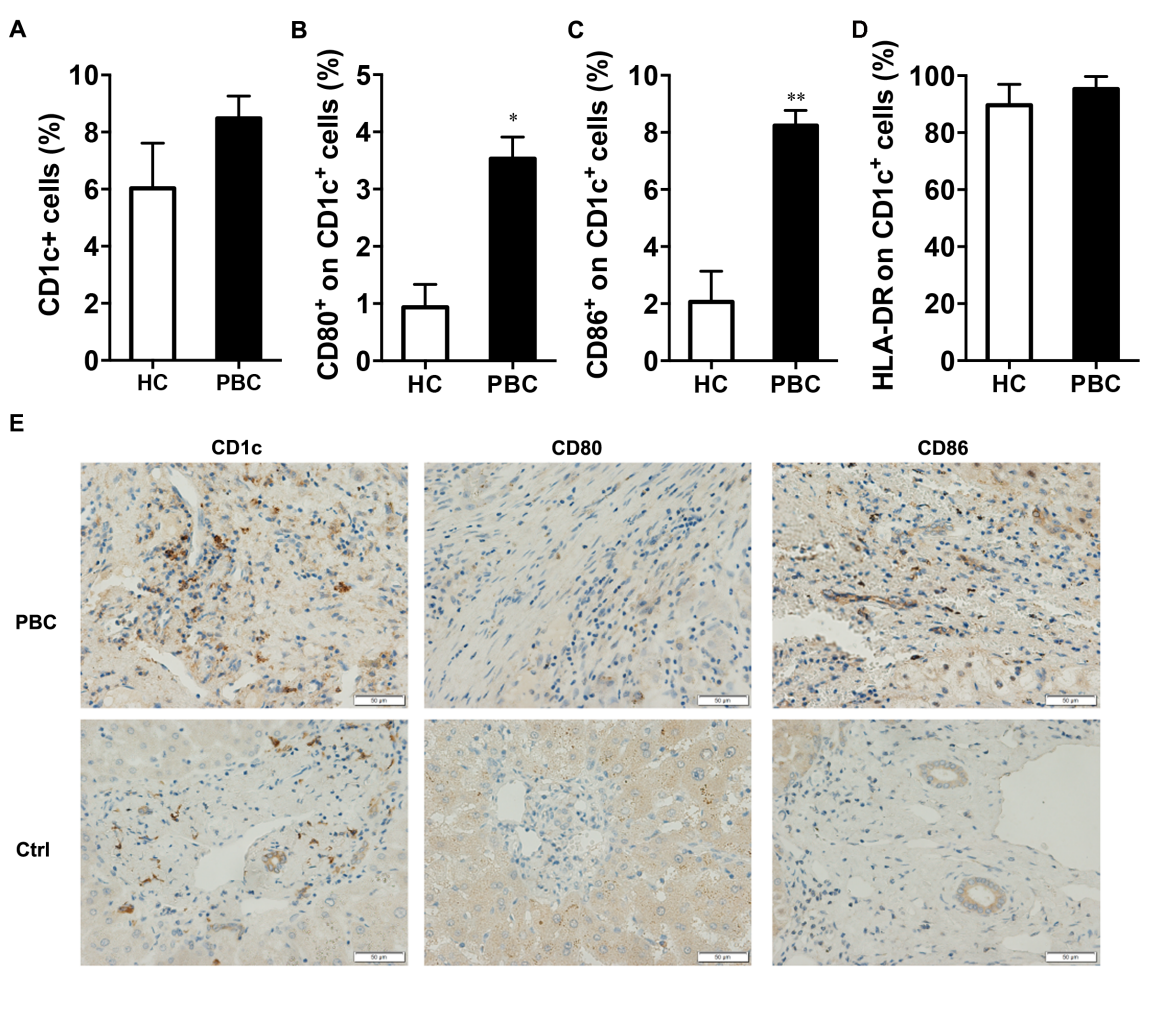
The co-stimulation overexpression in the PBC patients The comparison of (**A**) the proportion of CD1c^+^ cells and (**B**) the co-stimulation CD80, (**C**) CD86 and (**D**) HLA-DR expression on CD1c^+^ cells between the PBC and HC groups (*n* = 55 and *n* = 61) by flow cytometry. Mann-Whitney U test (A-D), Data shown are mean ± SD. *P < 0.05, **P < 0.01. (**E**) Liver immunohistochemical staining of CD1c, CD80 and CD86 in PBC and disease controls (400×). Representative staining images from patients with PBC and controls are shown.

Antigen presenting cells (APCs) promote their biological activity via potential molecules in the process including related cytokines and co-stimulatory molecules [14]. Since the number of CD1c^+^ cells was higher in PBC patients than the HCs, we investigated part of the APC subgroups. However, the PBC and HC groups were similar in the proportion of CD1c^+^ cells (Figure 3A). We also analyzed the proportion of two other populations, CD303^+^ and CD141^+^ cells, and found no difference between the patients and the HCs (Supplementary Figure 3). Furthermore, the co-stimulation molecules CD80 and CD86 on the CD1c^+^ cells of the PBC patients were both higher than that of the HCs (Figure 3B and 3C), though no difference in HLA-DR on the CD1c^+^ cells between the two groups (Figure 3D).

We further compared the co-stimulation expression in liver between the PBC patients and the controls. There showed no difference in hepatic CD1c^+^ or CD80^+^ cells between the two groups. However, we found in PBC patients there was an intense aggregation of CD86 positive cells around interlobular bile ducts, which were more than the controls (Figure 3E). In addition, CD1c^+^ cells seemed not to have a direct effect on liver fibrosis as no difference in α-SMA expression by LX-2 when cultured with CD1c^+^ cells from PBC patients and the HCs (Supplementary Figure 4A).

### Defective inhibition on co-stimulation by Tregs in PBC patients

**Figure 4:**
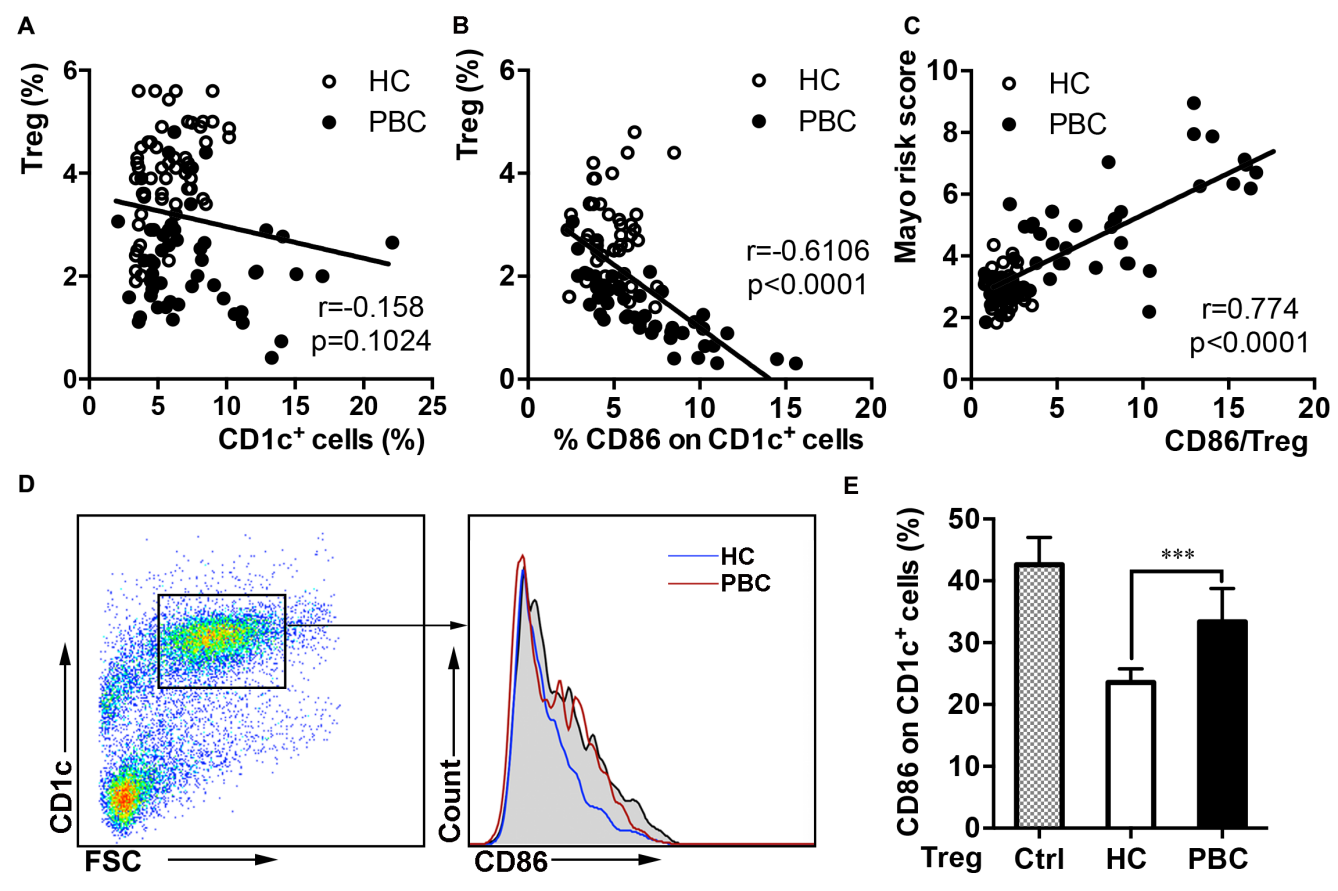
Less ability of Tregs to inhibit the co-stimulation expression on CD1c^+^ cells in the PBC patients (**A**) The proportion of Tregs compared with CD1c^+^ cells with Spearman’s correlation coefficients in the PBC patients (*n* = 55 and *n* = 61). (**B**) Correlation between the proportion of Tregs and CD86 expression on CD1c^+^ cells in the PBC and HC groups (*n* = 55 and *n* = 61). (**C**) Correlation between the ratio of CD86/Tregs and mayo risk score in the PBC and HC groups (*n* = 55 and *n* = 61). (**D**) CD86 expression on CD1c^+^ cells when cultured with or without (grey) Tregs from the PBC patients or HCs. (E) Statistical data (*n =* 8 per group). ANOVAs (**E**). Data shown are mean ± SD. *P < 0.05, **P < 0.01, ***P < 0.01.

As it was determined (above) that the Tregs of PBC patients were impaired and co-stimulation on CD1c^+^ cells was overexpressed, we investigated an association between Tregs and co-stimulation on CD1c^+^ cells. Interestingly, there was no correlation between the proportion of CD1c^+^ cells and Tregs in the PBC patients and HCs (Figure 4A), whereas a negative correlation was determined between the expression of CD86 on CD1c^+^ cells and Treg proportion in the enrolled individuals (Figure 4B). It has been reported that Treg-mediated downregulation of co-stimulation was crucial for inhibition of rapid T proliferation in mice, which directly influence the immune response after transplantation [10]. We also analyzed whether the prognosis is related to the ratio of CD86 and Tregs and found a positive correlation between mayo risk score and CD86/Treg (Figure 4C). It indicated the impaired inhibition on co-stimulation by Tregs could be relevant in PBC prognosis.

We next analyzed whether Tregs inhibited co-stimulation on APCs ineffectively in the PBC patients. We isolated Tregs from the PBC and the HC groups, and CD1c^+^ cells from the latter. The isolated Tregs from the two groups were each co-cultured for an *in vitro* suppression assay against HC CD1c^+^ cells, with concurrent incubation with inactivated *E.coli.* Interestingly, the Tregs from HCs, but not Tregs from the PBC patients, could significantly decrease the CD86 expression on CD1c^+^ cells (Figure 4D and 4E). IL-12, which could be naturally produced by APCs, is involved in the response to antigenic stimulation and IFN-γ production [15]. However, it showed no difference in IL-12 level (Supplementary Figure 4B) and CD80 expression on CD1c^+^ cells (Supplementary Figure 4C and 4D) in above *in vitro* suppression assay. This suggested that Tregs defect in suppressing CD86 expression might influence the progression of PBC.

### Effect of autoantibodies on liver fibrosis and co-stimulation in PBC patients

**Figure 5:**
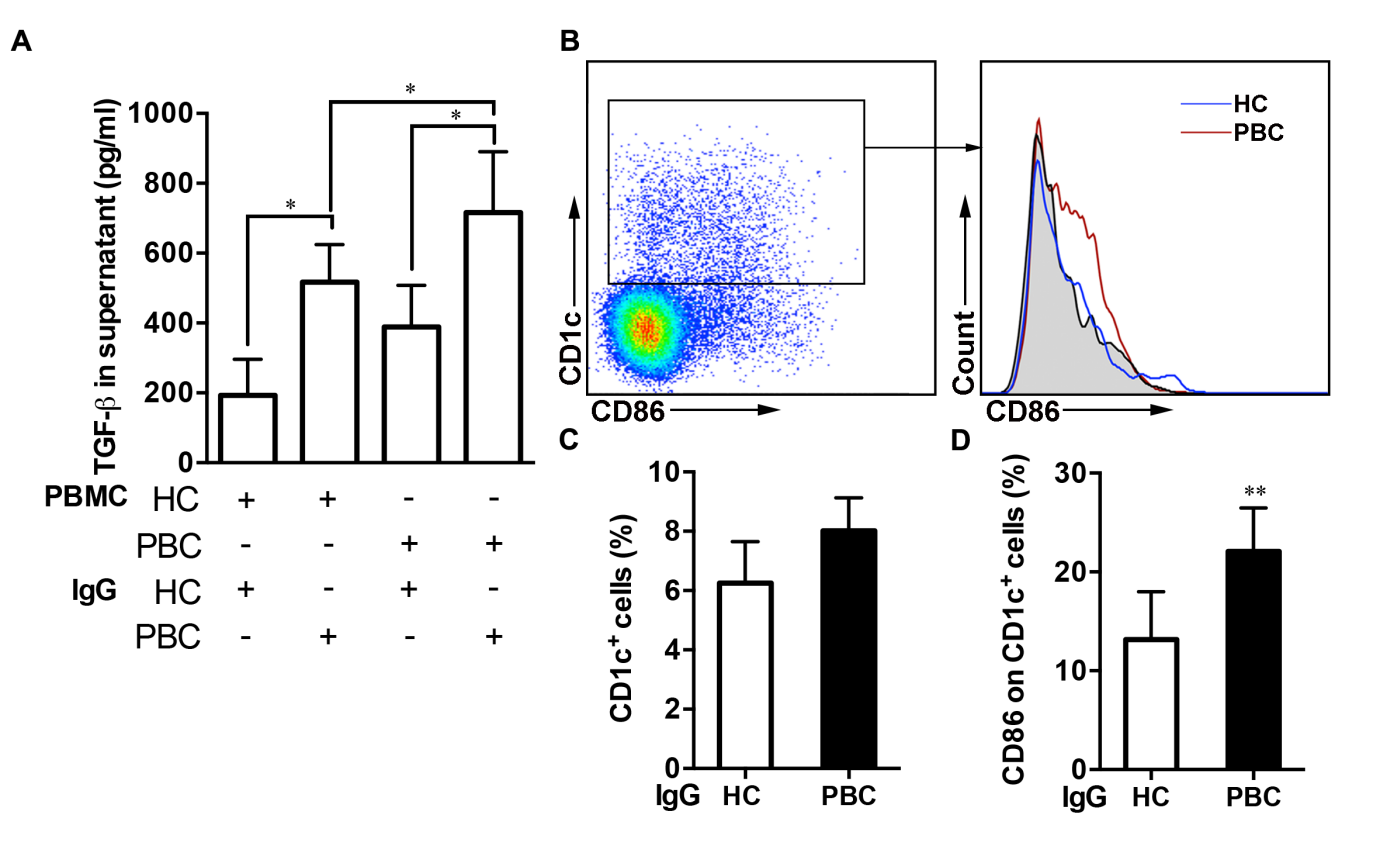
Self-antibodies promoted the progression of PBC (**A**) Comparison of TGF-β levels by PBMCs cultured with IgG in the supernatant. (**B**-**C**) The proportion of CD1c^+^ cells and (**D**) CD86 expression on CD1c^+^ cells was detected after PBMCs stimulated with or without (grey) IgG from the HCs and PBC patients by flow cytometry (*n =* 8 per group). ANOVAs (A), Unpaired Student’s *t* test (C-D). Data shown are mean ± SD. *P < 0.05, **P < 0.01.

Various autoantibodies may be positive during the progression of PBC. Thus, we treated the PBMCs from the two groups with IgG taken from each of these groups. When compared with PBMCs not IgG from the HCs, IL-10 levels were lower in the supernatant of that from the PBC patients (Supplementary Figure 5A). Furthermore, there was a higher level of TGF-β in the supernatant when human stellate cells were cultured with PBMCs or IgG from the PBC patients compared with from the HCs (Figure 5A).

Furthermore, we treated PBMCs of the HCs with IgG isolated from the two groups and found no significant difference in the proportion of CD1c^+^ cells after stimulation between the two groups (Figure 5B and 4C). However, the levels of CD86 on the CD1c^+^ cells with the IgG of the PBC patients were significantly higher compared to that of the HCs though no difference in the levels of CD80 on the CD1c^+^ cells between the two groups (Figure 5B - 5D, Supplementary Figure 5B and 5C).

IL-12 production by PBMCs after the IgG stimulation of the PBC patients in the supernatant was higher than that of the HC group (Supplementary Figure 5D). We further stimulated the isolated CD1c^+^ cells directly by IgG from two groups. It showed the higher CD86 expression on CD1c^+^ cells with IgG of PBC patients than that of the HCs, whereas no difference in CD80 expression on CD1c^+^ cells (Supplementary Figure 5E and 5F). This suggests excessive activation of CD1c^+^ cells by auto-antigenic stimulation in the progression of PBC.

## DISCUSSION

Tregs are reportedly impaired, in numbers or function, in autoimmune diseases such as type 1 diabetes, aplastic anemia, and systemic lupus erythematosus [6, 16-18]. Therapies that augment the numbers and function of Tregs have also been shown beneficial for the prevention of autoimmune diseases [19, 20]. Immunotherapy of regulatory T cells was even successfully applied in mice that suffered from autoimmune cholangitis [21].

Our present study primarily investigated the role of Tregs in the inhibition of T cell proliferation and co-stimulatory during the progression of PBC. We found that there were more apoptotic biliary epithelial cells and higher α-SMA levels of human hepatic stellate cells, which could be due to lower numbers of Tregs and impaired function of Tregs on suppression T cell proliferation. Furthermore, at the presence of autoantibodies, it showed a significant higher CD86 expression on CD1c^+^ cells, which could not be inhibited by the defective Tregs. Both of them contributed to the deterioration of PBC.

We found that the numbers and proportion of Tregs were both lower in PBC patients compared with the HC group, which is consistent with previous reports [22, 23]. The immunosuppressive cytokine IL-10 has also been implicated in activation of Tregs [24]. In the present study, compared with the HCs, IL-10 production by Tregs from the PBC patients was significantly lower. Tregs actively suppress activation of the immune system and prevent pathological self-reactivity [25]. Treg functional assays have indicated that Tregs from PBC patients insufficiently suppress the proliferation of CD4^+^ T cells and the IFN-γ level. That was also supported by the result about cytotoxicity of PBMCs against cholangiocytes. As there was a discrepancy between the present results of Treg function and the previous research, it could be due to the diverse definition of regulatory T cells [26]. Therefore, Tregs may have a dual role in the setting of obstructive jaundice, by suppressing T cell function while limiting cholestasis and hepatic fibrosis.

Kaji et al, found there more CD86 but not CD80 positive cells around interlobular bile ducts in liver of patients with PBC than the normal liver [27]. Our present results also showed that the expression of CD80 and CD86 on circulating CD1c^+^ cells was indeed significantly higher in the PBC patients than in the HCs, whereas only higher CD86 expression in the liver. The number of CD1c^+^ cells was increased in circulation rather than liver and no marked change in the proportion, which could result from the inflammation-induced slight increase of lymphocytes. As CD1c mainly expressed on the APCs including B cells and DCs, it indicated the disruption of the CTLA4-CD80/CD86-CD28 signaling in the interaction between APCs and T cells.

It was reported that IL-10 might have a crucial role in the progression of liver fibrosis [28, 29], which was also consistent with our present study. In the present study, there was a lower level of IL-10 in the supernatant when human stellate cells were treated with PBMCs from PBC patients than from the HCs. Furthermore, treatment with Tregs from the HCs could inhibit the α-SMA expression of human stellate cells, due to the higher level of IL-10 by Tregs in the regulation of fibrosis (Supplementary Figure 2). CD1c^+^ cells from the two groups did not influence α-SMA expression (Supplementary Figure 4A). This may reflect the function of deficient Treg regulation in PBC development.

As Tregs and CD1c^+^ cells made a difference simultaneously in the number, we investigated the pertinence of these two populations. There was no correlation between the proportions of CD1c^+^ cells and Tregs, whereas the CD86 expression on CD1c^+^ cells negatively correlated with the proportion of Tregs. Interestingly, there was also a positive correlation between the ratio of CD86/Treg and mayo risk score. Our finding on the importance of the co-stimulation/Treg ratio was consistent with published studies in a variety of models that indicate that a deficiency in induction of Tregs leads to PBC [10, 30]. Furthermore, An *in vitro* suppression assay against CD1c^+^ cells from HCs showed that the CD86 expression on CD1c^+^ cells could be significantly inhibited by Tregs from HCs, but little by Tregs from the PBC patients. This suggested that Tregs might regulate CD1c^+^ cells activation by suppressing CD86 expression.

In the normal liver, T cell abnormal activation may be suppressed via several mechanisms. In particular, liver T lymphocytes show a tolerogenic response to antigen-presenting cells [31]. Moreover, it was reported that Treg-depleting strategies come at a cost to T-cell receptor diversity and peripheral lymphocyte expansion, which enhanced the wrong recognition and response to self-antigens such as rheumatoid arthritis [32]. Therefore, self-antibodies in PBC could also be involved in this disease. In the present study, IgG from the PBC patients could have contributed to an increase in CD86 expression of CD1c^+^ cells; and the PBC patients were more capable of IL-12 production with stimuli. Also, both the PBMCs and self-antibodies from the patients may have contributed to the elevated level of TGF-β in the supernatant; the level of IL-10 in the supernatant was quantified and was lower in the PBMCs from the PBC patients compared with the HCs. Antigen presenting cells could induce T cell responses upon antigen uptake via Fcγ receptors[33]. And HSCs also are an important source of TGF-β. Our data showed IgG from PBC patients could increase the higher CD86 expression on CD1c^+^ cells, which could enhance the T cell response. Then, the enhanced T cell response contributes to HSCs producing such as collagen fibres as well as TGF-β via chronic liver injury and pro-inflammation and inflammation cytokines. This indicates that the autoimmune response could further promote the development of liver fibrosis.

The mechanism that controls the process of PBC and promotes treatment efficacy remains undetermined. Our results indicate that the above effects could readily be explained via Treg-dependent reduction in co-stimulation [34], thus limiting the abnormal activation of T cells. However, our finding showed Treg impairment in inhibition on T cell proliferation and co-stimulation, while Lan et al found there was no functional deficiency on PBC Tregs. This may due to region, race and disease severity of the enrolled individuals.Accumulating evidence suggests that Tregs are a dynamic population that can convert to IL-17- or IFN-γ-expressing T cells under certain conditions, such as with IL-12 stimuli. Also, the IL-23/Th17 pathway perpetuates IL-12/Th1-mediated immunopathology in PBC [35]. The conversion of Tregs to IFN-γ^+^ T cells has been reported in an autoimmune diabetic model as well as in a lethal infection model [36].

In summary, Treg impairment in number, IL-10 production, and inhibition of T cell proliferation may promote bile duct injury and liver fibrosis in PBC patients. Furthermore, co-stimulation overexpression also has an essential role in the progression of PBC. The correlation between Treg cell number and expression of CD86 leads us to speculate that Tregs have a crucial role in controlling the steady-state level of co-stimulation under physiological conditions. Tregs are similarly capable of mediating downregulation of CD86, which could provide full protection against PBC.

## MATERIALS AND METHODS

### Patients

Over 2 years, 55 consecutive patients received a diagnosis of PBC in our hospital. The study was approved by the Institutional Ethics Committee of First Affiliated Hospital, College of Medicine, Zhejiang University (Reference 2016-261). The work described has been carried out in accordance with The Code of Ethics of the World Medical Association (Declaration of Helsinki). Informed consent was obtained for experimentation with human subjects.

The criteria for inclusion in the study were the following: serum alkaline phosphatase (ALP) and γ-glutamyl transpeptidase (γ-GT), serum aminotransferase and bilirubin, conventional bile ducts on ultrasonography or other radiological examination, and serum-positivity for antimitochondrial M2 antibody. Patients who tested negative for antimitochondrial antibodies were accepted if they met all the other criteria for the diagnosis of PBC. In addition, the Mayo risk score was used for predicting survival in non-transplanted patients suffering from PBC, in an accordance with an approved mathematical model [37].

Patients who were confirmed with autoimmune disease overlap syndrome were excluded from this study. Patients with viral, obstructive, or metabolic etiologies or primary sclerosing cholangitis were also excluded, as well as those with drug-induced liver injury. Age- and gender-matched volunteer healthy control individuals (HCs) were also recruited at the hospital during the same period, after matching for demographic and other background data (Table 1).

### Flow cytometry

Peripheral blood mononuclear cells (PBMCs) were isolated using Ficoll. After centrifugation, cells were washed with PBS containing 0.5% bovine serum albumin, and the viability of cells was confirmed using trypan blue dye exclusion.

PBMCs were resuspended in staining buffer (0.5% bovine serum albumin, 0.04% ethylenediaminetetraacetic acid [EDTA], 0.05% sodium azide in PBS), washed and stained with the following for 30 min at 4 °C: anti-human peridinin chlorophyll (PerCP)-conjugated CD3/fluorescein isothiocyanate (FITC)-conjugated CD4/ phycoerythrin (PE)-conjugated CD8 (SK7, SK3, SK1); FITC-anti-human CD4 (RPA-T4); allophycocyanin (APC)- anti-human CD25 (M-A251); PE-anti-human CD127 (HIL-7R-M21); FITC-anti-human CD80 (L307.4); FITC-anti-human CD86 (FUN-1); FITC-anti-human human leukocyte antigen-antigen D related (HLA-DR, G46-6; BD Biosciences, San Diego, CA, all of the above); anti-human PE-CD16-56/FITC-CD3 (3G8, N901, UCHT1; Beckman Coulter, Brea, CA); PE-anti-human CD1c (AD5-8E7) and PE-anti-human invariant natural killer T (iNKT, 6B-11; Miltenyi Biotech, Auburn, CA). Stained cells were washed and fixed with 1% paraformaldehyde in PBS.

A FACS Canto II instrument (BD Immunocytometry Systems, San Jose, CA) was used for data acquisition. Data were analyzed with Diva-8 (BD Immunocytometry Systems) and FlowJo (Tree Star, Ashland, OR) software. The gain and gates set for analysis were identical for samples from PBC patients and HCs.

### Intracellular cytokine staining

PBMCs (2 ×10^6^/mL) were harvested, washed, stained with FITC–anti-human CD4 and APC-anti-human CD25, and then fixed using a Cytofix/Cytoperm Fixation/Permeabilization Kit (BD Biosciences, San Diego, CA) after stimulation with phorbol myristate acetate (50 ng/mL), ionomycin (1 µg/mL) and Golgi stop containing monesine (0.7 µL/mL) for 4 hours. Permeabilized cells were incubated for 30 min at room temperature with PE–anti-human interleukin (IL) -10 or isotype control (eBioscience, San Diego, CA). Stained cells were washed twice in permeabilization/wash buffer and resuspended in PBS supplemented with 0.3% w/v bovine serum albumin and 0.1% w/v sodium azide. The proportion of cytokine-expressing Tregs and T cells per 10^5^ cells was determined by flow cytometry.

### Cytotoxic assay

Isolated PBMCs were cultured overnight with recombinant human IL-2 (10 ng/mL). The target cells (RBE, human cholangiocarcinoma cells; cell bank of the Chinese Academy of Sciences) were labeled with carboxyfluorescein succinimidyl ester (CFSE; Molecular Probes, Eugene, OA) in advance to differentiate them from PBMCs, as we previously described [38]. The cells were harvested and seeded at an effector/target cell ratio of 10:1 for 12 or 24 hours. Cells were harvested and stained with propidium iodide (eBioscience, San Diego, CA) and the cytotoxic activity was assayed using flow cytometry.

### Histopathological analysis

Liver tissues were fixed in 10% neutral formaldehyde, embedded in paraffin, sectioned and then stained with hematoxylin-eosin (H&E). Immunohistochemistry staining for CD1c (ab156708), CD80 (ab134120) and CD86 (ab53004, all from Abcam, UK) were performed in formalin-fixed, paraffin-embedded liver sections according to the manufacturer’s instructions.

### Treg suppression assays

Enriched populations of T cells (CD4^+^CD25^–^) and Tregs (CD4^+^CD25^+^) and CD1c^+^ cells were separately isolated using a FACS Aria III instrument (BD Immunocytometry Systems, San Jose, CA) and cultured with IL-2 (10 ng/mL) overnight. The purity of the isolated T cells and Tregs were both greater than 95%. The enriched and CFSE-labeled T cells were either cultured alone at 2.5 × 10^5^ cells/well or cocultured at 2 × 10^5^ cells/well with a ratio of 4:1 (T:Tregs). Cultures were performed in 96-well round-bottom plates previously coated with either media (for control) or PMA (Sigma) at a concentration of 0.5 µg/mL. The cultures were then incubated at 37 °C for 4 days. The percent of Treg suppression was then calculated.

The enriched populations of CD1c^+^ cells were either cultured alone at 2 × 10^5^ cells/well or co-cultured with Tregs at 1 × 10^5^ cells/well with a ratio of 1:1 at the presence of inactivated *E.coli* (cell: bacteria=1:10). The cultures were then incubated at 37 °C for 12 hours. And CD86 expression on CD1c^+^ cells was detected by flow cytometry,

### Cytokine analysis

IL-10, interferon (IFN) -γ, IL-12, and transforming growth factor β (TGF-β) levels were measured using human enzyme-linked immunosorbent assay (ELISA) Ready-Set-Go Kits (eBioscience, San Diego, CA) in accordance with the manufacturer’s instructions. The cytokine content was expressed as amount per mL of plasma or supernatant.

### Isolation and purification of immunoglobulin G

Isolation and affinity purification of self-antibodies from serum samples of the PBC patients and HCs were conducted using Thermo Scientific NAb Protein G Spin Kits. Six to ten milligrams of IgG could be isolated and purified from 2 mL of the sera in accordance with the instructions. The IgG purification was over 95%. The PBMCs or CD1c^+^ cells were cultured at 1 × 10^6^ cells/mL with a concentration of 100 μg/mL.

### Real-time reverse transcription-PCR (RT-PCR)

Total RNA from cells was isolated using RNA Plus (Takara, Dalian, China) and cDNA was synthesized from 2.5 μg of RNA using a One Step PrimeScript RT-PCR Kit (Takara). Real-time PCR was monitored online using an ABI 7900 machine (Applied Biosystems, Foster City, CA) and SYBR Green master mix (Takara). The primer sequences were: GAPDH (glyceraldehyde-3-phosphate dehydrogenase) forward 5′-GGAGCGAGATCCCTCCAAAAT-3′ and reverse 5′-GGCTGTTGTCATACTTCTCATGG-3′; α-smooth-muscle actin (α-SMA) forward 5′-CTATGAGGGCTATGCCTTGCC-3′ and reverse 5′-GCTCAGCAGTAGTAACGAAGGA-3′. Relative quantification of special genes were normalized to GAPDH and calculated by the 2-ΔΔCT methods, where CT is the cycle threshold.

### Statistical analysis

Statistical analyses were performed using GraphPad Prism version 6.0 and SPSS 24.0 software. When comparing 2 groups, the Mann-Whitney U test or Student’s *t*-test was performed. Comparisons of multiple groups were performed using one-way analysis of variance with a Newman-Keuls post hoc test. Pearson’s correlation analysis was performed for normally distributed variables and Spearman’s rank correlation analysis was used for non-parametric variables. *P*-values < 0.05 were considered significant (* p < 0.05; ** p < 0.01; ***p<0.001).

## SUPPLEMENTARY MATERIALS FIGURES


