# Supplementary material for: Regulatory T cells with a defect in inhibition on co-stimulation deteriorated primary biliary cholangitis
Source: Oncotarget. 2017 Nov 26;8(65):108406–17. doi: 10.18632/oncotarget.22658 (PMC5752452; doi:10.18632/oncotarget.22658)
Supplement: Supplementary file 1 [file oncotarget-08-108406-s001.pdf]

Regulatory T cells with a defect in inhibition on co-stimulation deteriorated primary biliary cholangitis

SUPPLEMENTARY MATERIALS

Reagents and methods

(1) Sections from the controls and the patients with PBC were stained with hematoxylin and eosin (H&E) and examined; (2) PE-anti-human CD303 and PE-anti-human CD141 antibodies (BD Biosciences, San Diego, CA) were analyzed by flow cytometry; (3) The relative α-SMA expression was quantitated by q-PCR; (4) Peripheral blood mononuclear cells (PBMCs) or $CD1c^+$ cells from the HCs were isolated and then stimulated by IgG from the HCs and PBC patients. (5) The isolated Tregs (HC or PBC) were co-cultured with $CD1c^+$ cells (PBC) at the presence of inactivated *E.coli*. And CD80 expression on $CD1c^+$ cells was detected by flow cytometry, and the levels of IL-12 and IL-10 were both determined in the supernatant by ELISA.

Statistical analyses were performed using SPSS 24.0. When comparing two groups, the Mann-Whitney U test or Unpaired Student's *t* test was performed. Comparisons of multiple groups were performed using one-way analysis of variance with a Newman-Keuls post hoc test. Pearson's correlation analysis was performed for normally distributed variables and Spearman's rank correlation analysis. *P*-values < 0.05 were considered significant (*).

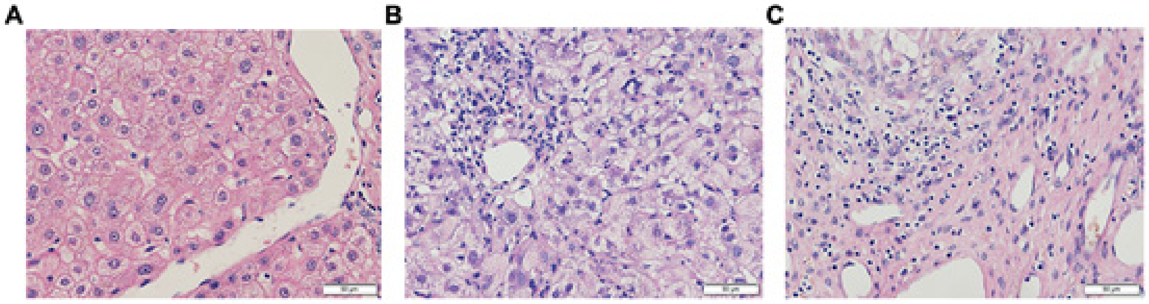

Supplementary Figure 1: Hepatic H&E sections and representative images of (A) the control and (B and C) PBC patients (400×).

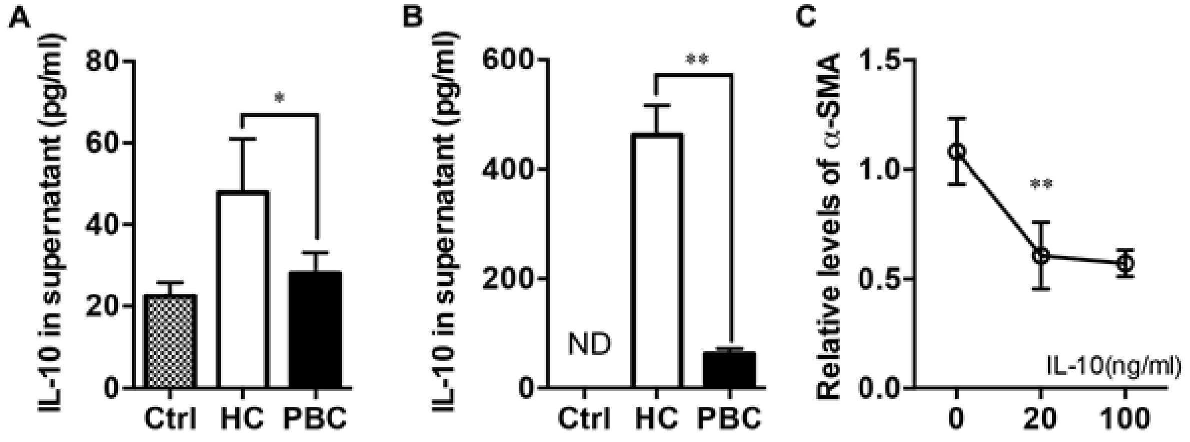

Supplementary Figure 2: IL-10-dependent inhibition on PBC. (**A**) The level of IL-10 in the supernatant was analyzed when CD4+ T cells were cultured with Tregs from the PBC and HC groups, respectively; (**B**) The level of IL-10 in the supernatant was accessed when LX-2 cultured with PBMCs from the PBC and HC groups;(**C**) α-SMA expression by LX-2 was detected with treatment of IL-10. Data shown are mean ± SD. Data shown are mean ± SD. ANOVAs. $*P < 0.05$, $**P < 0.01$.

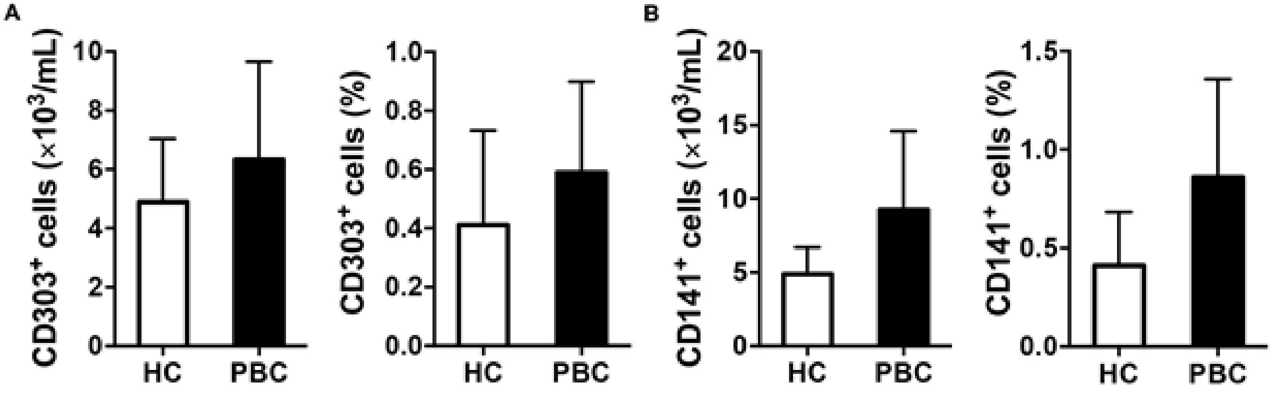

Supplementary Figure 3: Comparison of CD303+ and CD141+ cells in absolute numbers and the proportion between the PBC and HC groups. (**A**) CD303+ cells (**B**) CD141+ cells. Data shown are mean ± SD.

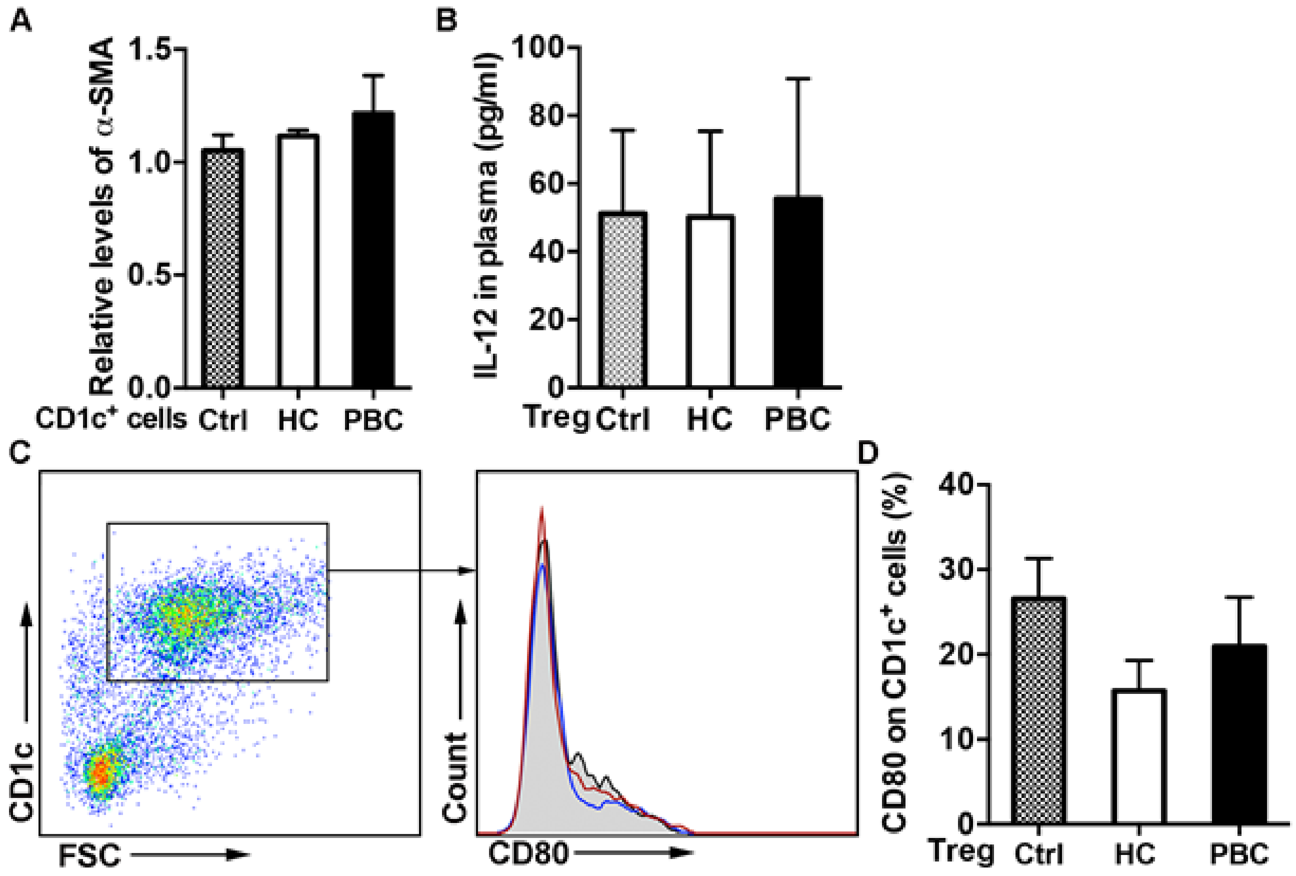

Supplementary Figure 4: The effect of Treg on IL-12 level and CD80 expression by CD1c$^+$ cells. (**A**) The expression of α-SMA in LX-2 cells was determined when cultured with CD1c$^+$ cells; (**B**) The level of IL-12 in the supernatant and (**C** and **D**) CD80 expression on CD1c$^+$ cells when cultured alone (grey) or co-cultured the isolated Tregs at the presence of inactivated *E.coli*, Data shown are mean ± SD.

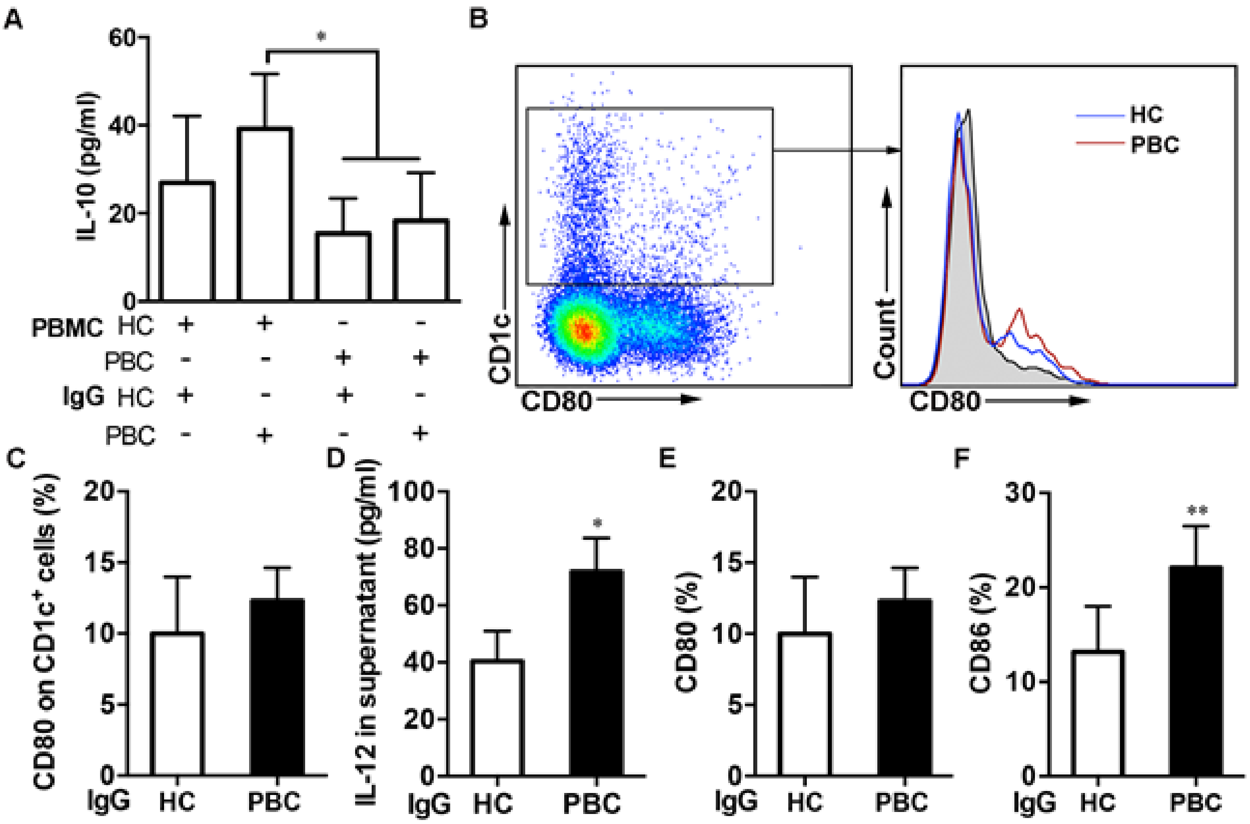

Supplementary Figure 5: The effect of autoantibodies on PBC. (**A**) Comparison of IL-10 levels by PBMCs in the supernatant at presence of IgG; (**B** - **C**) The CD80 expression on CD1c$^+$ cells was detected after PBMCs stimulated or not (grey) by IgG from the HCs and PBC patients, and (**D**) the level of IL-12 was determined in the supernatant. (**E** and **F**) The CD80 and CD86 expression on CD1c$^+$ cells was detected after the isolated CD1c$^+$ cells stimulated by IgG from the HCs and PBC patients. Data shown are mean ± SD. Unpaired Student's *t* test. $^*P < 0.05$, $^{**}P < 0.01$.
